# MK2 promotes p16 negative head and neck cancer migration, invasion, and metastasis

**DOI:** 10.1016/j.canlet.2025.217690

**Published:** 2025-04-02

**Authors:** Deri Morgan, Dakota DD. Okwuone, Kiersten L. Berggren, Levi Arnold, Alyssa Schmidt, Colby Spiess, Hannah Smith, Ravi Yada, Nathan Hendrikse, Rashna Madan, Devin Shrock, Chris Lominska, Mengjia Hu, Malgorzata Witek, Steven Soper, Yuting Lin, Hao Gao, Dennis J. McCance, Sufi M. Thomas, David Beebe, Sheena C. Kerr, Gregory N. Gan

**Affiliations:** a Department of Radiation Oncology, University of Kansas Medical Center, Kansas City, KS, USA; b The University of New Mexico, School of Medicine, Albuquerque, NM, USA; c Carbone Cancer Center, University of Wisconsin Madison, WI, USA; d Department of Pathology, University of Kansas Medical Center, Kansas City, KS, USA; e Department of Chemistry, University of Kansas, Topeka, KS, USA; f Department of Pathology, The University of New Mexico, Albuquerque, NM, USA; g Department of Otolaryngology, University of Kansas Medical Center, Kansas City, KS, USA

## Abstract

For patients with locally advanced, p16-negative head and neck squamous cell carcinoma (HNSCC), overall survival remains poor due to primary locoregional failure and distant metastasis following curative therapy. We aimed to understand how MAPKAPK2 (MK2) regulates HNSCC tumor cell migration and invasion, important first steps in cancer metastases. The TCGA database and HNSCC tissue microarrays were used to show that MK2 expression was associated with more advanced cancers and faster cancer recurrence rates. We observed that silencing of tumor MK2 in human cell lines (shRNA) caused a significant reduction in tumor cell migration-invasion in a complex HNSCC microphysiologic system used to recapitulate the tumor microenvironment. Murine cells (Ly2) with MK2 silenced (CRISPR-Cas9) also demonstrated reduced migration and invasion using 2D and 3D monoculture cell migration-invasions assays. Ly2 cells are orthotopic p16-negative murine metastatic cells that spontaneously metastasize, and we observed that MK2 inhibition via genetic (Cas9/CRISPR) or pharmacologic (PF-3644022) methods led to a significant reduction in the number of circulating tumor cells, fewer lymph node and lung metastases, and MK2 inhibited mice showed improved overall survival. Our findings suggest that HNSCC MK2 regulates tumor cell migration-invasion and may be a promising therapeutic target to reduce metastases.

## 1. Introduction

For patients with p16-negative head and neck squamous cell carcinoma (HNSCC), locoregional disease recurrence with or without distant metastatic disease remains a substantial problem impacting disease control and long-term survival [1]. Attempts to escalate therapy for locally advanced disease by adding biotherapy [2], induction chemotherapy [3,4], radiation intensification [5], and immunotherapy [6] over standard of care cisplatin-radiotherapy alone have failed to improve progression-free survival, overall survival, and distant metastases-free survival rates. Treatment options for patients who have locoregionally recurrent disease following definitive chemoradiotherapy is limited to salvage surgery in the best performers or re-irradiation when surgical salvage is not an option [7]. Two-year survival outcomes for these two populations are approximately 60 % and 35–40 %, respectively [7,8]. In cases of recurrent/distant metastatic disease, current first-line therapy consists of either chemotherapy combined with immunotherapy or immunotherapy alone, with 2-year overall survival rates around 29.4 % and 27 %, respectively, for non-combined probability score stratified patients [9].

The efficacy of locoregional therapy is reduced due to regional or distant metastatic disease. This is underscored by the fact that primary management of early-stage and locally advanced HNSCC relies heavily on either surgery and/or radiotherapy (with or without chemotherapy) as curative first line treatment. Fundamentally, cancer locoregional recurrence and distant metastases requires activation of cellular migration and invasion pathways. Treatments that target tumor cell migration-invasion cell signaling could reduce patient tumor dissemination and metastasis to the cervical lymph nodes, mediastinum and lung, which are commonly the first sites for metastases in HNSCC patients [1,10].

MAPKAPK2 (or MK2) is a stress response protein regulated (via phosphorylation) by p38α MAPK. MK2 regulates gene expression, post-transcriptional gene regulation and interacts with regulatory proteins involved in autophagy, cell motility, invasion, and inflammation that may contribute to the metastatic cascade [11,12]. In HNSCC the action of MK2 is thought to enhance proliferation and metastasis through long coding mRNA [13], transcript stability [14] and cross talk of genes such as IGFBP2, MUC2 and PRKAR3B [15]. We have previously demonstrated that MK2 activation in HNSCC contributes to radiotherapy resistance and high MK2 phosphorylation in p16-negative oropharyngeal HNSCC patients was a poor prognostic feature associated with significantly worse disease specific survival compared to patients with low MK2 phosphorylation. MK2 inhibition (PF-3644022) significantly suppressed growth of a patient derived tumor in nude mice [16]. Yet, the effect of tumor MK2 on the progression of HNSCC from local to distant disease is not established. Here we demonstrate that MK2 suppression affects migration, invasion, and metastasis of HNSCC.

## 2. Methods

Full scientific methods and techniques are available in the supplemental methods section.

### 2.1. The Cancer Genome Atlas data analysis

RNA sequencing data for head and neck squamous cell carcinoma (HNSC) were accessed through cBioPortal from The Cancer Genome Atlas (TCGA) Firehose Legacy cohort [20,21]. MK2 expression levels were analyzed based on gene-level transcript quantification, which was log-transformed as log2(x+1) and normalized using RSEM counts. Expression values were then correlated with available clinical data from TCGA, including disease stage, lymph node involvement, perineural invasion (PNI), extracapsular spread, angiolymphatic invasion, p16 (HPV) status, sex, race, and age. Statistical analyses were performed using unpaired t-tests for two-group comparisons and one-way ANOVA for multi-group analyses, with a significance threshold set at *P* < 0.05.

### 2.2. LumeNEXT fabrication

Fabrication of the PDMS microdevice was performed as previously described using standard soft lithography techniques [17]. Briefly, polydimethylsiloxane (PDMS, Dow Corning, Sylgard 184) mixed 10:1 was poured over the SU-8 silicon master molds and used to fill 25-gauge (Fisher Scientific, 14–840-84) hypodermic needles. PDMS components were then baked at 95 °C for 1 h. After baking, the 260 μm PDMS rods were extracted from the needles and placed between the two layers. The devices were adhered to a glass-bottom MatTek dish (MatTek Corporation, P50G-1.5–30-F) and UV sterilized for 20 min before use.

### 2.3. Microphysiologic tumor spheroid invasion assay

PDMS devices were treated with 2 % poly(ethyleneimine) (PEI, Sigma-Aldrich) in water for 10 min followed by 0·4 % glutaraldehyde (GA, Sigma-Aldrich, in water) for 30 min. Devices were washed with water and dried. High-density rat-tail collagen type 1 (Corning), 10X PBS was neutralized with 0.5 M NaOH (Fisher Scientific) to pH 7·4. A final 3 mg/mL concentration of collagen was obtained by adding fibronectin, 100 μg/μL (Sigma-Aldrich), and fibroblast media (ScienCell). Primary head and neck patient fibroblasts were isolated from residual tissue acquired from surgery with informed consent under an approved Institutional Review Board protocol at the University of Wisconsin, Madison as previously described [17]. Fibroblasts were added at a final concentration of 500 cells/μL. Cal27 or Tu-167 cells were stained with Vybrant DiD (ThermoFisher) at 1:1000. Tumor spheroids were formed using the hanging drop method previously described [17]. After 48 h tumor spheroids were transferred to the ECM solution. 7 μL of the ECM solution was loaded and polymerized at room temperature for 30 min. PDMS rods were removed to create a lumen. Human lymphatic endothelial cells (HLECs, ScienCell) were added into the lumen at 20,000 cells/μL (3.5 μL total) and incubated at 37 °C for 40 min. Devices were washed with endothelial media (ScienCell) and cultured with 10 μL of endothelial cell media that was replenished daily. Devices were imaged at day 1 and day 4 using a Nikon TI^®^ Eclipse inverted microscope. Z-stacks were collected at each time point. Images were analyzed using NIH ImageJ software where the z-stack was z-projected and thresholded. A region of interest (ROI) was drawn around the spheroids at day 4 to capture the maximal distance of migration. The area fraction of the cells was calculated in the ROI between matched day 1 and day 4 images. Percent change in area fraction quantified cancer cell migration and was compared across conditions as previously described [17].

### 2.4. Migration-invasion assays

#### For Scratch Migration assays:

220,000 Ly2 Cells were plated into the wells of 24 well plates Cells were allowed to adhere overnight and washed the following day with 1x PBS. Wells were scratched with a thin line down the middle of each well using a p20 pipette tip and non-adherent cells washed and aspirated off and fresh serum-free media re-added. Cells were imaged immediately following scratch (0 h) and then again at 24 and 48 h. Pictures were evaluated by comparing 0-h to 24- and 48-h closure of the scratch gap. Pictures were analyzed using ImageJ and differences were statistically analyzed using GraphPad Prism.

#### For Scratch Invasion Assays:

Adapted from Ref. [22], the scratch migration protocol was followed with the addition of a 200 μL layer of 3 mg/mL Matrigel on top of the scratched cells, allowing 30 min to polymerize @ 37 °C.

#### Spheroid Dissemination Assay:

Assay was adapted from a previously described protocol with slight modifications [34]. 500 single cells were added to the wells of 96-well ultra-low attachment plates (96-well Clear Round Bottom Ultra-Low Attachment Microplate, Corning, 7007). After 72 h, the resulting spheroids were transferred into a 96-well cell culture-treated plate, and dissemination of the cells from the spheroid was measured as the fold change in surface area covered by the cells relative to the time of transfer (0-h time point) as measured by ImageJ software at 40× magnification.

#### Spheroid Invasion Assay:

Assay was adapted from a previously published protocol, with minor modification [35]. Forty 20 μL droplets of 500 cells were placed on the lid of a Petri dish, and the lid was inverted onto the bottom of the dish, which had 5 mL of distilled water to prevent dehydration. Droplets were given 72 h to form spheroids and were then collected in a microcentrifuge tube and allowed to settle. 40 μL of the settled spheroid were mixed in 200 μL of 3 mg/mL Matrigel, and 40 μL of the resulting mix was promptly added to the center of the wells a 24-well plate. These 3D cultures were given at least 30 min to polymerize and then covered with complete media. Invasion was measured by the change in area covered by the cells of individual spheroids relative to the start of the experiment (0-h time point) as measured by ImageJ software at 40× magnification.

### 2.5. Mouse studies

All animal experiments were approved by the University of Kansas Medical Center IACUC. Female BALBc and NSG mice were kept in 12-h dark 12-h light cycles with unlimited access to food and water. For orthotopic implants, mice were anesthetized with isoflurane gas and 50,000 Ly2 cells were implanted with a 26.5-gauge needle in a volume of 50 μl (50 % Matrigel (Corning), 50 % DMEM) to the floor of mouth with the needle accessing the tissue though the open mouth and to the side of the tongue. Mice were left to their devices and monitored 3x a week for tumor growth. Tumor growth was measured by electronic calipers (Fine Science Tools). When tumors were above 500 mm^3^ mice were given diet gel to help calorie intake. To dose the mice with the MK2 inhibitor, PF-3644022, 50 μl of 2.6 mM PF-3644022 solution in PBS was injected into the mice 3 times a week (final concentration = 1.8 mg/kg or ~5 μM, half-life ~ 9hrs [18]). Mice were euthanized at experiment end, if the tumor volume exceeded 1000 mm^3^ or if the mice displayed morbidity or excessive weight loss (20 % of bodyweight). Kaplan Meyer survival curves were generated comparing WT to KO cells with survival being measured from day of implant onwards by Prism 10 (GraphPad Software). Mice were euthanized by CO_2_ asphyxiation (5 %) until breathing ceased for at least a minute. Blood was taken via cardiac puncture before continuing with post-mortem necropsy. Tumors, lungs and lymph nodes were isolated during necropsy and placed into 4 % paraformaldehyde for 24 h. After 24 h the samples were placed in 70 % ethanol until processing. Formalin-fixed paraffin-embedded samples were serially sectioned at 5 μm thickness which included primary tumors, lymph nodes, and whole lung, and stained with hematoxylin and eosin. Histological sections were prepared using a standard protocol, stained with hematoxylin and eosin (H&E) and cover slipped with permanent mounting medium. H&E-stained slides were evaluated by a board-certified pathologist using light microscopy to identify and quantify the extent of metastatic involvement of lung and lymph node tissue.

### 2.6. CTC analysis

Ly2 tumor cells express both FAPα and EpCAM by Flow cytometry (Supplemental Fig. 3B). Blood from each mouse was collected via cardiac puncture and stored in EDTA to prevent coagulation. Blood was then introduced into a serially sequenced microfluidics platform coated with capture antibodies (FAPα or EpCAM) [19], we collected fractions of circulating tumor cells expressing EpCAM and FAPα, in the blood of Balb/c mice orthotopically engrafted with parental Ly2 tumors at endpoint (Supplemental Fig. 3C). Tumors were immunophenotyped for PanCK and Vimentin expression using immunofluorescence (Supplemental Fig. 3D) and enumerated.

### 2.7. Statistics

Unless otherwise noted, data is expressed as mean ± SEM. For pairwise comparison of data sets, pre-planned Student’s t-test comparisons (Prism 10, GraphPad software) were performed for both *in vitro* and *in vivo* experiments. All *in vitro* experiments were replicated at a minimum of three separate experiments. *In vivo* animal experiments were statistically analyzed among groups with 7–10 animals per group. Statistical values were only shown for *p* < 0.05.

## 3. Results

### 3.1. MK2 regulates human head and neck cancer migration and invasion

**Fig. 1.**
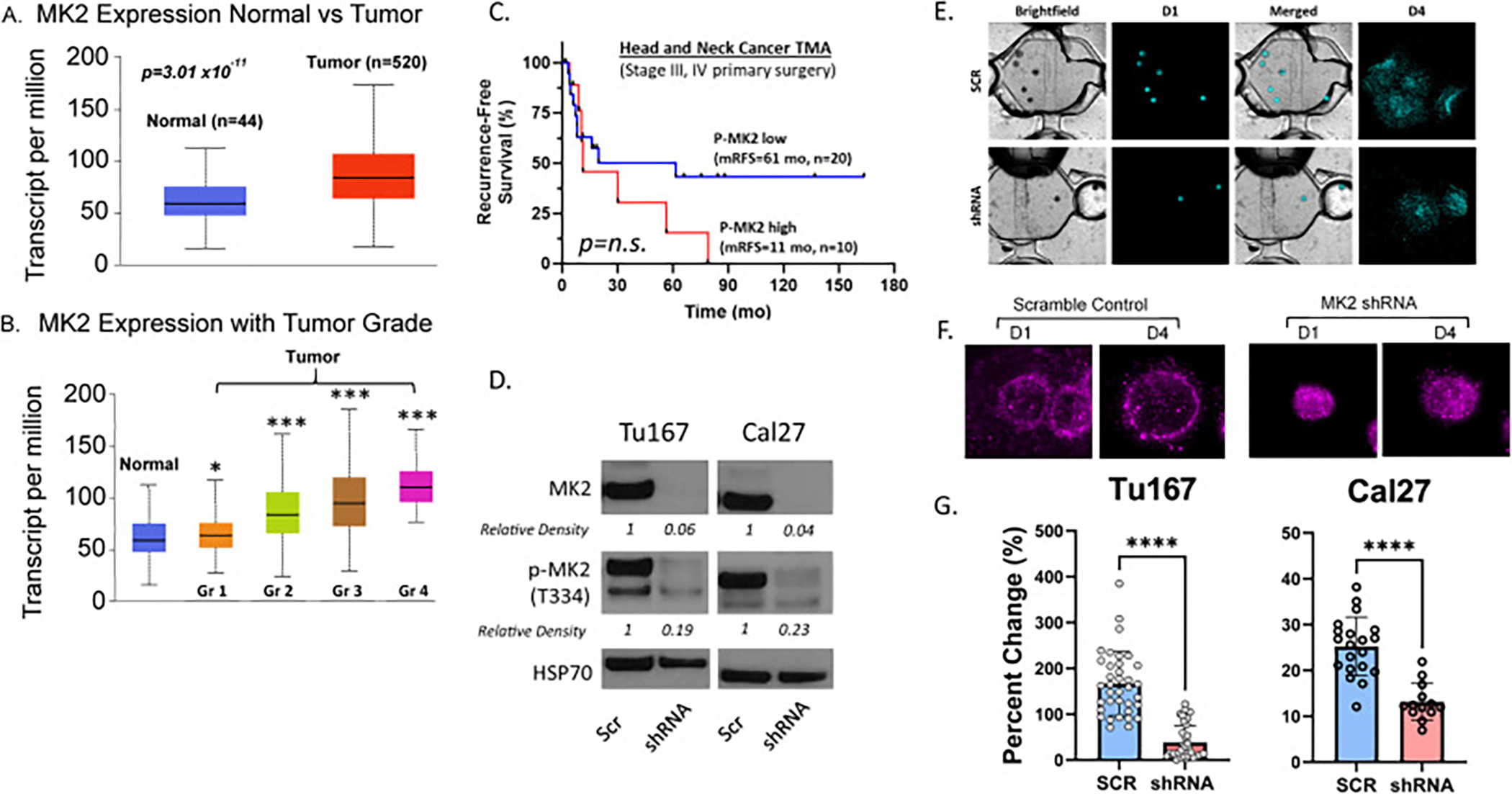
Loss of tumor MK2 leads to reduced tumor cell migration and invasion. **A, B.** We performed a TCGA analysis of 520 head and neck cancer specimens with 44 normal tissue control specimens and evaluated MK2 transcript expression between normal and tumor specimens. MK2 transcript levels were stratified based on normal tissue and increasing tumor grade. **C.** Immunohistochemistry (IHC) of phospho-MK2 (p-MK2) was performed on a head and neck cancer tissue microarray (TMA) derived from HPV-negative, p16-negative oropharyngeal squamous cell carcinoma patients undergoing primary surgery. Kaplan-Meier recurrence-free survival analysis performed on the median MK2 phosphorylation. Slides were digitally acquired using Aperio platform and objective quantification of IHC staining performed using HALO. **D.** SDS-PAGE was performed on RIPA-lysed cell extracts from scramble and shRNA cell lines followed by immunoblot. **E.** MK2 scramble and shRNA HNSCC cell line spheroids (Tu167, Cal27) were grown in an MPS (LumeNEXT) populated by human cancer-associated fibroblasts and lymphatic endothelial vasculature. Cells were pre-stained with Vybrant DiD and migration was evaluated on day 1 (d1) and d4. The extent of tumor cells migration (% cellular area change) was compared between days. **F** Close up view of Tu167 scramble and shRNA at d1 and d4 demonstrating extent of tumor migration-spread in the MPS. **G.** Quantification of tumor cell migration. A minimum of 3 independent experiments were performed with multiple biologic replicates as shown for Tu167 and Cal27. Statistical analysis performed. P-values: * <0.05, ** <0.01, *** <0.001, **** <0.0001.

Using the TCGA, we found that head and neck cancer patients have higher MK2 gene expression compared to non-cancer patients and MK2 transcript levels increased with worsening tumor grade (Fig. 1A and B). We conducted additional analyses examining MK2 expression across key clinical variables, including age, HPV (p16) status, and sex, and found no significant differences in expression based on these factors. This suggests that MK2 expression is broadly distributed among diverse patient subgroups rather than being driven by these demographic or clinical characteristics (Supplemental Figs. 1A–D). As locoregional recurrence remains a significant problem in HNSCC, we analyzed a surgically resected p16-negative oropharynx tissue microarray and found that high MK2 phosphorylation (p-MK2) compared to low MK2 phosphorylation trended towards worse recurrence-free survival (mRFS 11 vs 61 mo, p = n.s., HR 0.5789, 95 % confidence interval 0.203–1.653) (Fig. 1C). Local and regional disease dissemination requires the ability to migrate and/or invade into the surrounding microenvironment. To this end, we developed MK2 shRNA constructs in 2 human HNSCC models (Cal27 and Tu167) that demonstrated reduced MK2 expression and phosphorylation (Fig. 1D) to assess MK2’s role in migratory-invasive capacity. We had previously observed alterations in EMT gene expression levels in our MK2 inhibited Tu167 and Cal27 cell lines and examined how MK2 suppression impacted common HNSCC EMT protein expression (Supplemental Fig. 4) [16]. While MK2 inhibition can lead to reduced EMT protein levels overall, there are differences in which EMT protein is reduced observed between Tu167 and Cal27. Stromal cells (i.e., cancer associated fibroblasts, endothelial cells) can contribute to tumor cell migration and invasion [22–24]; therefore, we employed an established, 3D microphysiologic system (MPS) [25–29] to evaluate MK2-driven invasion in a more physiologically relevant multicellular tumor microenvironment. The MPS included shRNA MK2 tumor spheroids or scramble control embedded in a collagen-fibronectin matrix that contained primary human cancer associated fibroblasts and a microvessel seeded with human endothelial cells. We saw that control tumor spheroids showed extensive invasion into the microenvironment while loss of MK2 reduced the ability of these cells to spread (Fig. 1E and F) resulting in significantly lower invasion (Fig. 1G; Tu167 = 167 % ± 11.7 % (scr) v 38.2 % ± 6.1 % (shRNA); Cal27 = 25 % ± 1.4 % (scr) v 13 % ± 1.1 % (shRNA)). Taken together, these data show that MK2 knockdown decreases tumor cell invasion compared to MK2 WT tumor cells. Furthermore, differences in cell migration-invasion were not attributable to changes in cell proliferation as there were no differences in the proliferation of control vs shRNA cells (Supplemental Fig. 2A and B).

### 3.2. Loss of MK2 leads to reduced migration-invasion and tumor metastases in metastatic murine models

**Fig. 2.**
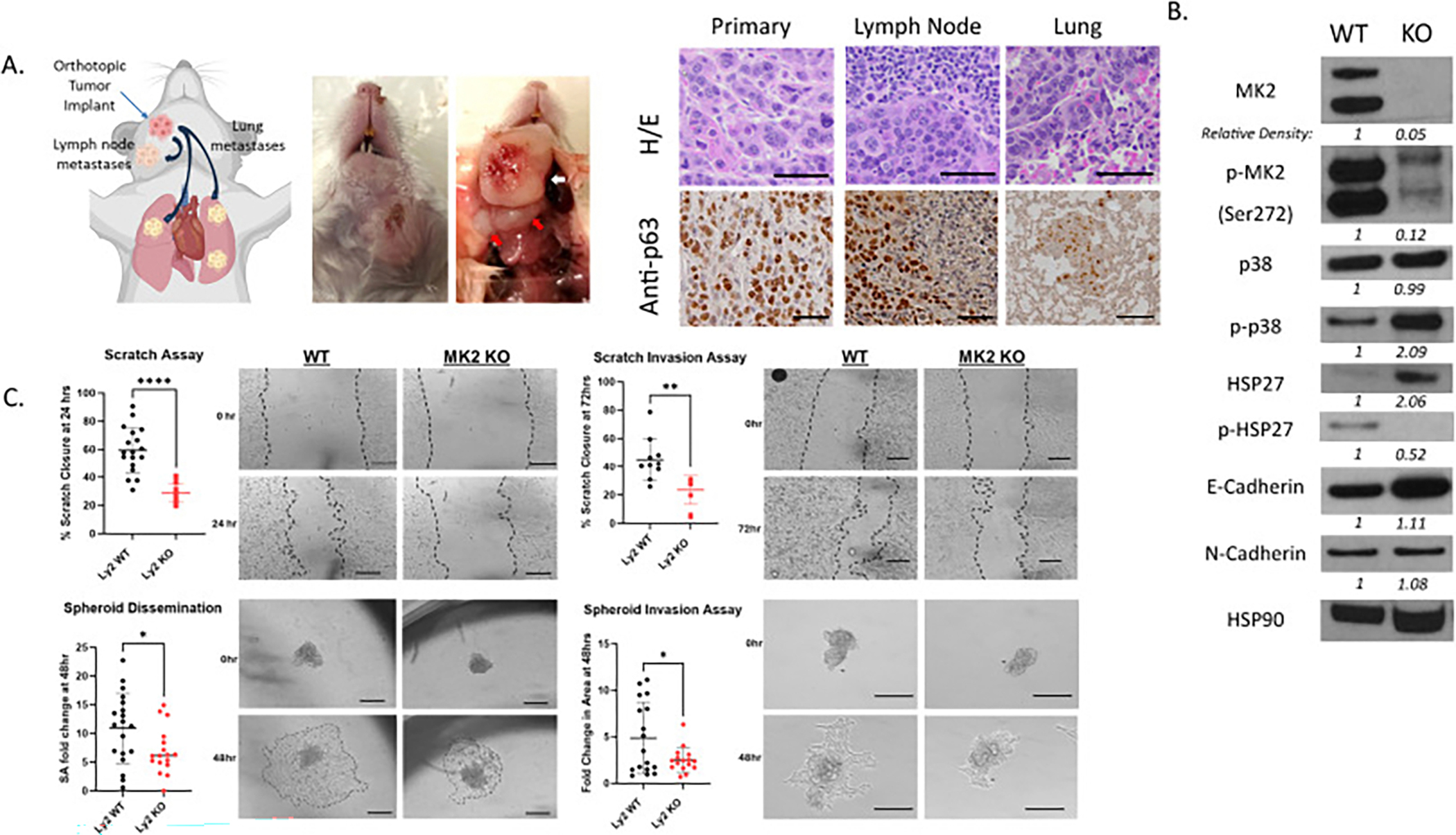
Loss of tumor specific MK2 in murine Ly2 tumor cell migration and invasion. **A**. Representative figure demonstrating natural HNSCC progression from primary tumor to lymph node and lung following orthotopic tumor implantation into the floor of mouth. Image developed using BioRender. The murine Ly2 tumor cell line was orthotopic implanted into the floor of mouth (FoM) of Balb/c mice. White arrows denote primary HNSCC tumor and red arrows highlight peri-tumoral lymph nodes. Representative H/E and anti-p40 stained primary, lymph node and lung metastatic tumors. **B.** Ly2 parental wild type (WT) and Cas9/CRISPR MK2 knockout (KO) cell lines were immunoblotted for p38-MK2-HSP27 pathway proteins and mesenchymal-epithelial markers. **C**. Scratch migration and invasion assays were performed to evaluate tumor cell migration and invasion using a 2D method. Spheroid dissemination and spheroid invasion assays were performed to evaluate tumor cell migration and invasion using a 3D method.

The Ly2 HNSCC model is ideal for investigating mechanisms of metastasis as an orthotopic floor-of-the-mouth implantation into syngeneic mice (Balb/c) allows for natural tumor metastases to the lymph nodes and then the lung (p63 staining identifies squamous cell) which recapitulates the human pathobiology (Fig. 2A). CRISPR-Cas9 was used to knockout functional MK2 genes by frameshift mutation and clones were confirmed by genomic sequencing. Loss of MK2 was demonstrated by absence of MK2 expression and HSP27 phosphorylation by immunoblot (Fig. 2B). Whereas p38 and N-cadherin expression remained constant across both cells, increased E-Cadherin was seen in the MK2 knockout (KO) cell lines compared to the parental wild type. We hypothesized that loss of tumoral MK2 would similarly impact the ability of our murine HNSCC tumor cell to migrate and invade into its surrounding environment. Migration measured using a traditional 2D scratch, a 3D spheroid dissemination assay, and invasion using a 2D scratch invasion or a 3D spheroid invasion assay all showed significant reductions in the ability of MK2 KO cells to migrate and invade (Fig. 2C). Furthermore, loss of MK2 did not impact cellular proliferation in these cells (Supplemental Fig. 1C).

**Fig. 3.**
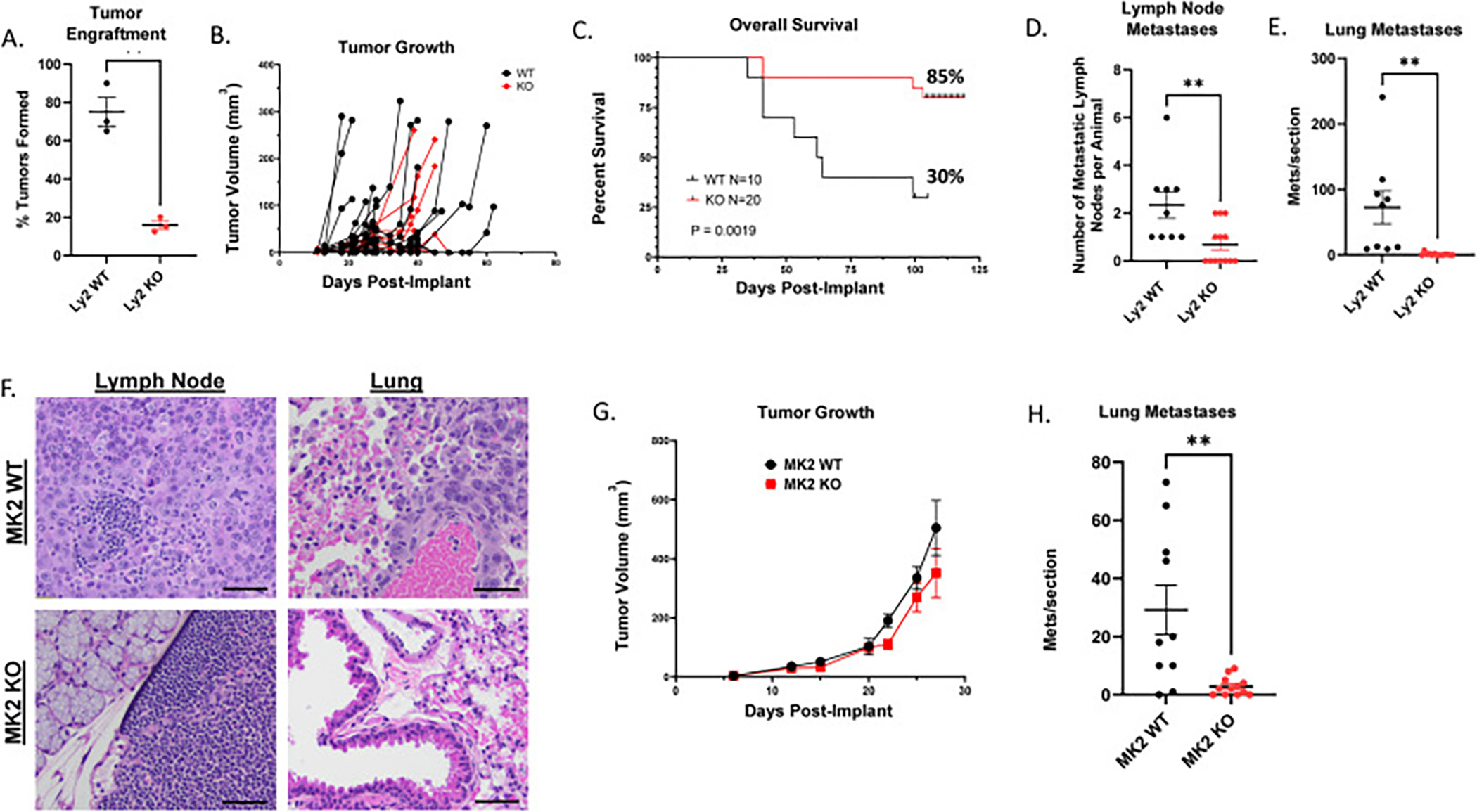
Loss of tumor MK2 reduces tumorigenesis as well as impairs distant lymph node and lung metastases, *in vivo*. **A** MK2 parental and KO Ly2 cells were orthotopically implanted into Balb/c mice using 50,000 cell transoral injection into the FoM. **B** Orthotopic tumor engraftment into immunocompetent Balb/c mice and tumor size was directly compared between WT and MK2 KO tumors. Tumor volumetric measurements were performed three times per week. **C.** A Kaplan Meier survival study was performed comparing orthotopically implant MK2 WT vs KO tumors. Survival was evaluated in all implanted animals. **D.** Total number of metastatic lymph nodes per animal were enumerated. WT, n = 9; KO, n = 14. **E.** Similarly, all 5 lung lobes were uniformly evaluated for number of metastases per mouse. **F.** Representative images from MK2 WT tumors and MK2 KO tumors were obtained at 20X magnification for lymph node and lung. **G.** MK2 WT and KO tumor growth in NSG mice evaluated by tumor volume measured by calipers. **G**. Total Lung sections were performed like **E** and total number of lung metastases were compared between MK2 WT and KO tumors. Statistical analysis performed. P-values: * <0.05, ** <0.01, *** <0.001, **** <0.0001.

When Ly2 parental and MK2 knockout tumors were orthotopically implanted into syngeneic mice (Balb/c), the MK2 KO cells demonstrated significantly lower tumor engraftment rates and tumor growth in 3 independent orthotopic xenograft studies with parental-wild type MK2 tumors (17 % vs 75 %, p = 0.0017) (Fig. 3A and B). Loss of tumor MK2 contributed to an improvement in overall survival (day100 OS: 85 % vs 30 %, p = 0.0019, Fig. 3C). We also observed a significant reduction in overall mean lymph node (2.3 vs 0.7, p = 0.0063) and lung (72.6 vs 1.1, p = 0.0026) metastases in the MK2 knockout tumor implanted mice compared to mice with Ly2 parental WT tumors (Fig. 3D and E). MK2 KO Ly2 tumors trended towards fewer circulating tumor cells (CTCs) compared to WT Ly2 tumors (Supplemental Fig. 3A). Representative images of lymph nodes and lungs taken from mice sacrificed on the same day, show Ly2 WT tumors exhibited higher rates of tumor infiltration into the external capsule of the lymph node followed by replacement and effacement of the node (Fig. 3F). Similarly, lung metastases were observed throughout lung parenchyma and in proximity to blood vessels of the lung (Fig. 3F).

We examined whether orthotopic tumor implantation into a NOD SCID IL2R-Gamma-null (NSG), immunodeficient mouse model, could impact tumorigenesis and metastases. Primary tumor growth rates were the same between MK2 WT and MK2 KO tumors (Fig. 3G). While loss of tumor MK2 did not impact tumorigenesis in immunocompromised mice, we observed a persistent significant reduction in mean lung tumor metastases from 29 to 3, p = 0.0029 (Fig. 3H).

### 3.3. MK2 inhibition with PF-3644022 leads to reduced head and neck cancer metastases

**Fig. 4.**
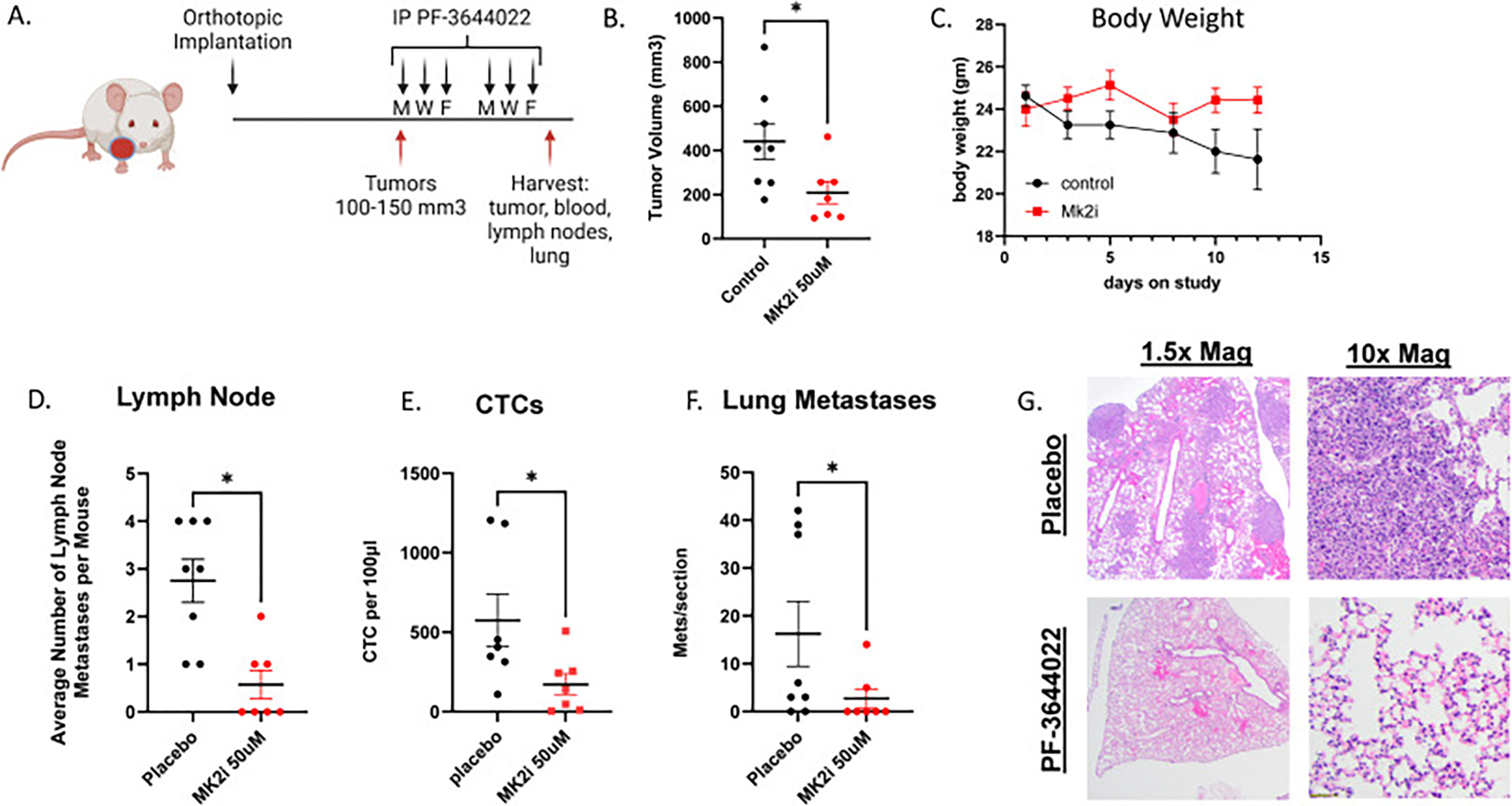
MK2 inhibition leads to reduced circulating tumor cells in orthotopically implanted Ly2 tumors. **A.** WT parental Ly2 tumors were orthotopically implanted into the FoM of Balb/c mice. When tumors were ~100 mm^3^, animals were treated with excipient control (placebo) or 50 μM PF-3644022 via IP injection. Drug was delivered every other day 3x/week for 2 weeks. Tumor volume was measured three times per week. Image created with BioRender.com. **B.** On the day of euthanasia, tumors size was measured one final time. Treatment with MK2 inhibitor led to a significant reduction in tumor volume. Animals were euthanized by CO2 asphyxiation followed by terminal exsanguination via cardiac puncture and primary tumors and lungs were harvested for evaluation. **C.** Body weight was measured with each treatment. **D.** Tumor and surrounding lymph nodes removed were evaluated for extent of metastatic disease. **E.** Blood was collected in EDTA tubes and stored on ice. Samples were passed through anti-FAPα coated and anti-EpCAM coated microfluidics devices in the KUMC BME Core Facility, washed in isotonic solution, and then FAPα and EpCAM expressing CTCs were eluted and immunophenotyped. Total number of EpCAM and FAPα expressing CTCs were enumerated between treatment arms. **F.** Lung metastases were evaluated from H/E-stained sections derived FFPE lung specimens from each treatment group. G. Representative images from a placebo drug treated and PF-3644022 treated mice. H/E stains performed to assess lung metastases. Student t-test performed. P value * <0.05.

We have previously shown in human HNSCC cell lines that inhibition of MK2 with the MK2 inhibitor, PF-3644022, could reduce EMT gene expression, *in vitro* [30]. Because of this, we investigated whether PF-3644022 would suppress tumor growth, metastasis, as well as formation of CTCs in our syngeneic metastatic model (Fig. 4A). Treating Ly2 WT tumors with 5 μM PF-3644022 (as previously published [30,31], Supplementary Fig. 2D shows LD50 for Ly2 cells at 11 μM) led to a significant reduction in orthotopic tumor growth compared to vehicle control-treated mice (Fig. 4B, p = 0.02). Mice in the MK2 inhibitor arm had more consistent weight compared to the placebo arm which began to experience increasing weight loss during the treatment period (Fig. 4C). Animals were euthanized at a fixed end point and tumor; lymph node and lungs were collected. Intracardiac puncture was also performed to collect CTCs allowing for an assessment of overall metastasis. In the vehicle-treated mice, we observed significantly more tumor-infiltrated lymph node metastases (Fig. 4D and 2.4 vs 0.8, p = 0.01), more numeric circulating tumor cells (Fig. 4E and 572 vs 172, p = 0.04), and more lung metastases (Fig. 4F and 16 vs 3, p = 0.05) compared to MK2 inhibitor-treated animals. Representative H/E images were taken at 1.5x and 10x magnification comparing vehicle and PF-3644022 treatment showing reduced/no metastases in the PF-3644022 treated mice.

## 4. Discussion

Loco-regional recurrence and distant metastasis remains a significant problem in patients treated with definitive chemoradiotherapy in non-surgically operable disease or in patients wanting an organ-sparing approach. We have previously reported that high MK2 phosphorylation was a poor prognostic factor associated with worse patient survival, radiotherapy could increase EMT gene expression and tumor cell MK2 inhibition could abrogate this effect, and combinatorial therapy in our *in vivo* human HNSCC PDX models could enhance local tumor control rates and improve overall survival [30]. Various teams have reported that EMT expression (i.e., Snail, Slug, Twist) alters kinetics in pre-clinical HNSCC *in vitro* migration-invasion studies [32–36] and retrospective patient studies have demonstrated an association of Snail and Slug expression with regional and/or distant cancer progression and chemoradiotherapy resistance [37–39]. However, our initial work lacked studies evaluating the role of tumor MK2 on HNSCC migration-invasion and metastases. To our knowledge, this study is the first to demonstrate how intrinsic tumor MK2 in HNSCC is implicated in tumor cell migration and invasion and its relevance to orthotopic tumorigenesis and spontaneous metastases in an animal model. This was accomplished using a combination of 2D/3D systems with human and murine HNSCC cell lines and a murine HNSCC tumor model capable of spontaneously metastasizing to the lymph nodes and lung in both immune-competent and immunodeficient animal models. Drug inhibition of parental Ly2 tumors orthotopically implanted into immunocompetent mice with MK2 inhibitor PF-3644022 resulted in decreased tumor volume, reduced numbers of CTCs, and reduced lymph node and lung metastases. The concentration used was consistent with the concentration of the effects of PF-3644022 used to inhibit TNFα release in mice [40]. However, while we cannot rule out off target effects, the concentration of drug used is above the LD50 of cell death when we perform our cell kill assays (Supplementary Fig. 2D). This work implicates that targeting the MK2 pathway in HNSCC can lead to reduced tumor metastases via inhibition of tumor cell migration-invasion. An upcoming Phase Ib/II clinical trial (NCT06374459) will evaluate the effect of the MK2 inhibitor, ATI-450, in hormone receptor positive, Her2-negative metastatic breast cancer and represents the first implementation of MK2 inhibition in the metastatic setting. We have previously demonstrated in a patient-derived xenograft model that a combination of MK2 inhibition and radiotherapy led to improved overall tumor control and survival. Our findings suggest that targeting MK2 alone while beneficial in reducing tumor dissemination, did not completely eliminate tumor growth in MK2 inhibitor treated tumors or the MK2 KO tumors. Because of this, local therapy (radiotherapy, surgery) combined with MK2 inhibition to suppress locoregional and distant metastases will likely remain necessary for long-term tumor control and survival.

Others have examined how systemic MK2 is thought to impact distant metastases in breast cancer. Murali et al. [41], found that tumor MK2 knockdown was unable to inhibit breast adenocarcinoma visceral and bone metastasis, *in vivo*. Using a systemically delivered MK2 inhibitor, they could reduce both visceral and bone metastases and improve mouse survival. How MK2 suppression in the tumor cells themselves versus stromal cells may differentially impact tumor progression remains unclear. Another group, using a MMTV-Her2 breast cancer model, showed that Her2 mediated suppression of the p38-MK2-HSP27 signaling axis facilitated early tumor cell dissemination and metastases through activation of the β-catenin pathway. Furthermore, pharmacologic MK2 inhibition led to cancer metastases, *in vivo* [42]. These findings are contrary to our own possibly due to organ specifics.

We observe the loss of tumoral MK2 impacts HNSCC tumor cell migration-invasion and metastases both *in vitro* and *in vivo*. The immunocompetent Balb/c mouse model showed loss of tumor MK2 led to significant reduction in tumorigenesis and a decrease in both lymph node and lung metastases. Using NSG mice, we eliminated the lymphoid mediated immune response which led to MK2 KO tumor growth rates comparable to the WT MK2 tumors. However, what was surprising was that loss of tumor MK2 demonstrated a persistent reduction in overall lung metastases, *in vivo*. When taken in the context of MK2 not effecting proliferation in the tumor cells, it is evident in the immunocompetent model that the immune system is playing a role in the difference in growth between WT and MK2 KO tumors. Nevertheless, the data from the NSG model suggests that MK2 regulates tumor metastases by means separate from pure tumor growth. These findings could be attributable to differences in histology (squamous cell carcinoma versus adenocarcinoma). The mechanism of MK2 on migration and invasion is not clear. We previously postulated that MK2 was affecting epithelial-to mesenchymal-transition (EMT) [16] but our data examining EMT genes across multiple HNSCC cell types do not show a consistent pattern of reduced expression of SNAIL and SLUG proteins (Supplementary Fig. 4). Whereas EMT seems disordered with MK2 inhibition it may not be the primary driving mechanism. A deeper mechanistic exploration is required to uncover how MK2 inhibition is affecting metastasis.

A limitation of our work is its focus on HNSCC biology as these findings could have broader relevance to other SCC histology such as cervical, anal, lung, and esophageal cancers. Further, we also do not understand how the long-term effects of this treatment might affect recurrence or how well this drug may be tolerated. The goal of our initial work using multiple models was to demonstrate for the first time that targeting MK2 in HNSCC could reduce specific markers of regional and distant metastases compared to non-treated (or control) animals. Further work is essential to reveal the feasibility of translating this potential treatment into the clinic. Regarding the tolerability of this drug, we may be able to extrapolate from prior reported experiences where MK2 knockout mice live normal full lives and are more resistant to developing inflammatory bowel disease [40] and colon cancer [16,31] following local chemical irritant treatment using azoxymethane/dextran sodium sulfate. Both models demonstrated a reduction in localized and systemic inflammation following chemical irritant along with MK2 inhibition using drug or by whole animal genetic MK2 knockout. Potential reduction in tumor or treatment associated inflammation may be one explanation for the observed reduced tumorigenesis and improved outcome. While we have focused on MK2 in this study, other pathways may also contribute to this process. Additional future studies that focus on global expression profiling to determine how other pathways interact with MK2 would be useful.

However, a strength of our findings is the use of both established human HNSCC cell lines, the use of a murine syngeneic metastatic HNSCC model and the use of both genetic as well as pharmacologic inhibition which demonstrates consistent findings that targeting the MK2 pathway can impact tumor cell migration, invasion, and metastases.

In conclusion, this study provides novel evidence demonstrating the relevance of intrinsic HNSCC MK2 expression in promoting tumor cell migration, invasion, circulating tumor cell formation, and metastases using a combination of genetic and pharmacologic models. This work provides evidence for the therapeutic potential of targeting MK2, in combination with localized therapy, for p16-negative HNSCC patients with or at risk of recurrent and metastatic disease.

## Supplementary Material

Multimedia component 1

Appendix A. Supplementary data

Supplementary data to this article can be found online at https://doi.org/10.1016/j.canlet.2025.217690.
