# Supplementary material for: MK2 promotes p16 negative head and neck cancer migration, invasion, and metastasis
Source: Cancer Lett. Author manuscript; Available in PMC 2025 Aug 3. (PMC12318363; doi:10.1016/j.canlet.2025.217690)
Supplement: Multimedia component 1 [file NIHMS2095537-supplement-Multimedia_component_1.pdf]

Supplemental Materials and Methods

Cell Culture

Human HNSCC cell lines Tu167 was kindly gifted from Dr Jeffrey Myers (MDACC) and Cal27 was obtained from ATCC repository. Both Tu167 and Cal27 underwent MK2 gene suppression via shRNA (with corresponding scramble control) using a commercial pre-packaged lentiviral (Origene) approach. GFP expressing cells were isolated and further selected using puromycin antibiotic. Individual MK2 knockdown clones were tested for MK2 suppression compared to scramble control via immunoblot. All human cells were incubated in DMEM with 5 mM L-glutamine containing 10 % fetal bovine serum (FBS) (Life Technologies), 100 U/ml penicillin-streptomycin (P/S) (Sigma-Aldrich). Ly2 was kindly gifted by Paola Vermeer (Sanford Health). Ly2 parental and MK2 knockout cell lines were generated using a Cas9/CRISPR method in collaboration with the KUMC Transgenic Mouse Core facility. Individual clones were evaluated by immunoblot for loss of MK2 expression between clones and parental. All subsequent clones used in experimentation were further exome sequenced to identify the region of mutational stop codon. Ly2 was cultured in 1:1 DMEM with 5mM L-glutamine and HAM's F12 media supplemented with 10 % FBS and 100 U/ml P/S. All cells were maintained at 37 ºC and 5 % CO_2.

Antibodies

All monoclonal and polyclonal antibodies for human and mouse studies were obtained from Cell Signaling Technologies. The following were used; MK2 (#12155), pMK2 T334 (#3007, Lot 6), HSP27 (#50353), pHSP27 (#9709), p38 (#8690), p-p38 (#4511), E-cadherin, (#3195), N-cadherin (#13116), Snail (#3879, Lot 14), Slug (#9585, Lot 7), Twist (#90445, Lot1), Vimentin (#5741), HSP70 (#4872, Lot 4), HSP90 (#4877). Secondary antibodies (Anti-Rabbit IgG #7074) were obtained from Cell Signaling Technologies.

Human Primary Tissue Microarray

As previously described [13], we examined a de-identified 180-patient oropharyngeal SCC TMA obtained from the Northern Ireland Biobank (NIB 13-001), with study approval in the United States under the UNM HRRC 15-565. Based on the UICC/AJCC 7th edition, we excluded low risk HNSCC patients who had Stage I or Stage II disease or did not receive definitive surgery. The remaining patients were defined as loco-regionally advanced (Stage III, IVA, IVB) p16-negative with a smoking history leaving 30 appropriate patients. TMA slides were stained with a p-MK2 antibody (Cell Signaling Technologies, Danvers, MA) by the HTR-TASR. Stained slides were digitally scanned and acquired on an Aperio 2AT scanner. Predetermined conditions were coded into HALO system (Indica Labs, Corrales, NM) by a blinded pathologist who helped with identifying staining intensity, percent staining of total tissue and identifying tumor cells from surrounding tissue microenvironment cells. All samples in the TMA were then subjected to machine-based image analysis. Results for nuclear staining intensity (0, 1+, 2+, 3+) and percent tumor tissue staining were obtained. Hirsch score (H-score) (intensity × percent staining) was performed for all samples. The median H-score was used as the cutoff value to classify tissue as high versus low p-MK2 staining. Log-rank statistical analysis was performed to assess recurrence free survival as a function of H-index. We acknowledge the generous contribution of Queen's University, Belfast, for providing us the tissue microarray and de-identified survival outcomes for the TMA. We also wish to acknowledge the University of New Mexico Comprehensive Cancer Center for access to their pathology tissue core facility.

Cell Proliferation and Kill Curve of PF3644022

Cell proliferation and dose response kill curve was measured using CyQuant. From cell culture, the cells were detached using 0.05 % Trypsin and plated in a sterile 96 ell plate at 10,000 cells per well and allowed to adhere overnight. The following day, the cells were treated with appropriate treatment along with a media change for the control wells. The 0 hr control plate was pulled at the time of treatment addition. Subsequent plates were then harvested every 24 hours after that. For the kill curve, cells were left in presence of the drug for 48 hours. Harvesting of a plate included, dumping the treatment media, washing each well with cold PBS, and then placing the plate in -80 C to freeze until every time plate had been harvested. Once all plates were harvested, they were removed from the freezer and allowed to thaw for 30 min – 1 hour. When fully thawed, 200 uL of CyQUANT GR dye/cell-lysis buffer was add to each well and then taken immediately to a Tecan Infinite M200 microplate reader. Sample fluorescence was read at 480 nm excitation and 520 nm emission maxima. LD 50 for PF3644022 was 11 μM.

Immunoblot

Cells were grown to 60-80% confluency and processed for protein. Briefly, cells were mechanically lysed with RIPA buffer with protease phosphatase inhibitors (Pierce) and a rubber scraper. Total protein lysates were quantified via standard Bradford assay using a Tecan Infinite M200. A total of 25 µg of protein extracts were loaded and separated using SDS-PAGE gel electrophoresis and transferred to PVDF membranes. Immunoblot was performed as previously described above and blots were examined with HSP70 and HSP90 as loading controls. Relative Density was calculated by measuring the density (via ImageJ) of the blotted protein divided by the density of the loading control and made relative to the WT or Scramble control.

Statistics

Unless otherwise noted, data are expressed as mean ± SEM. For pairwise comparison of data sets, pre-planned Student's t-test comparisons (Prism 10, GraphPad software) was performed for both *in vitro* and *in vivo* experiments. All *in vitro* experiments were replicated at a minimum of three separate experiments. *In vivo* animal experiments were statistically analyzed among groups with 7-10 animals per group. Statistical values were only shown for $p < 0.05$.

Supplemental Figure 1.

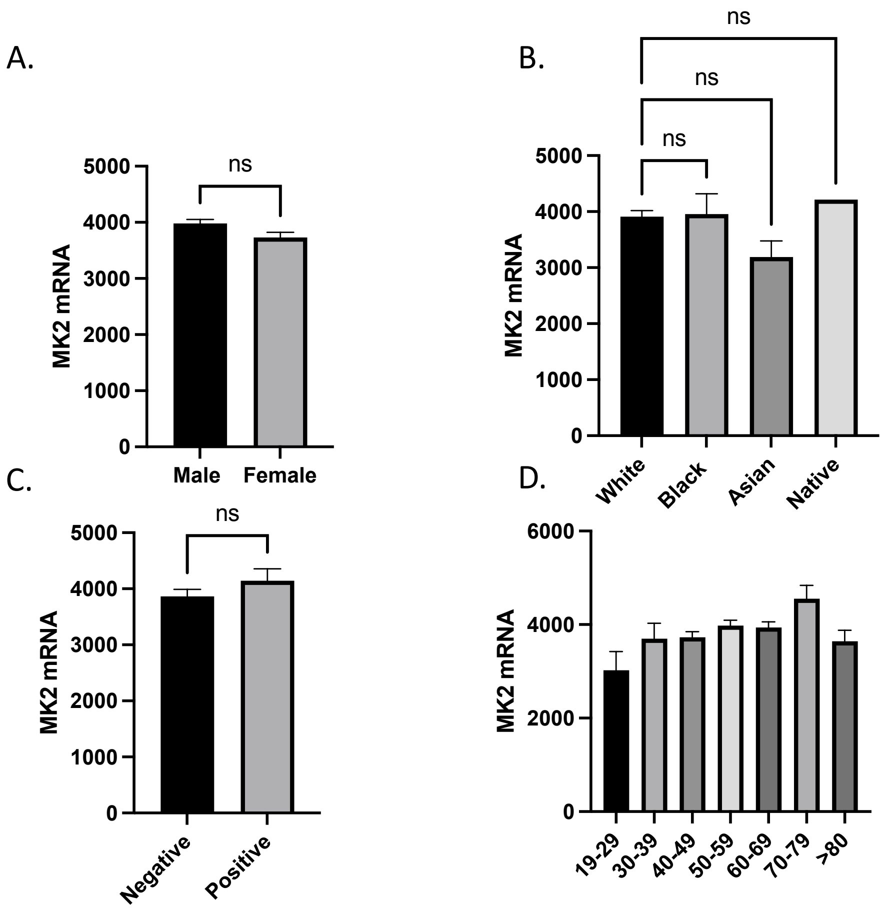

Supplementary Figure 1. MK2 gene expression in TCGA head and neck squamous cell carcinoma (HNSC) cohort across clinical and demographic variables. Bar graphs represent MK2 mRNA expression levels (RSEM-normalized log2 values) across various patient subgroups. Statistically significant differences were determined using unpaired t-tests or one-way ANOVA. Comparisons without asterisks were not statistically significant, and some multi-group comparisons contained too many data points for statistical annotations. No significant differences were observed based on A. sex, B. race C. p16 (HPV) status or D. age. Data were obtained from The Cancer Genome Atlas (TCGA) via cBioPortal.

Supplemental Figure 2.

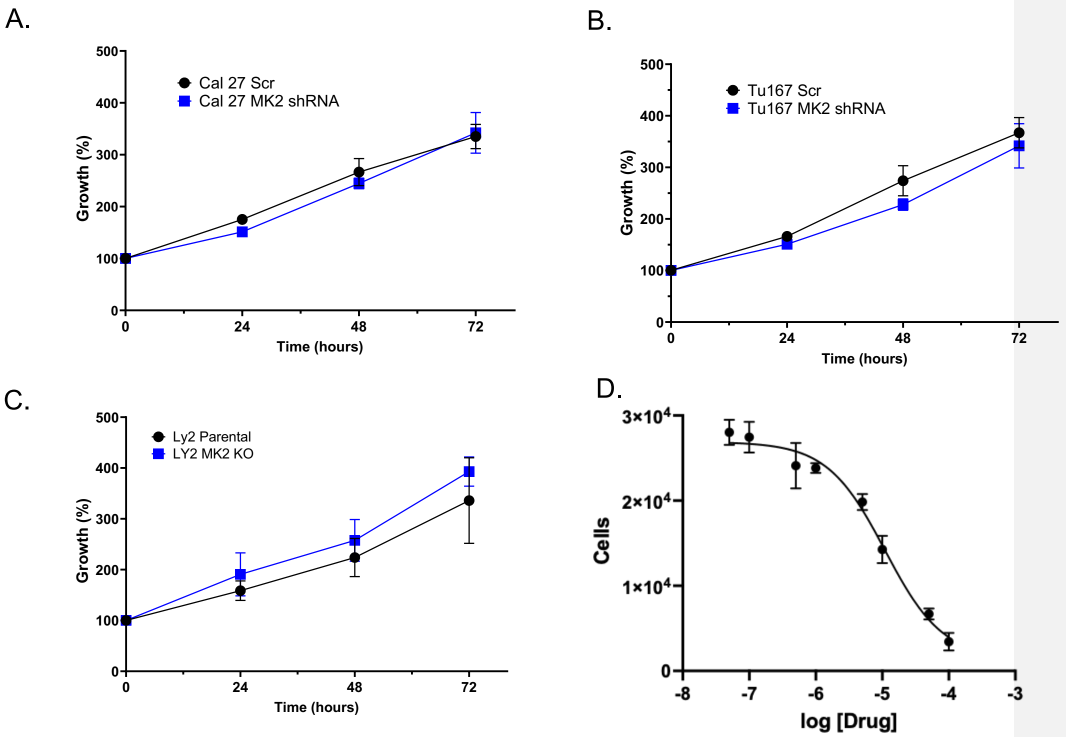

Supplemental Figure 2. Role of MK2 on HNSCC tumor cell proliferation. Tumor cell proliferation for **A.** Cal27 and **B.** Tu167 were measured via Cyquant fluorescence assay. Cells were collected at 24, 48 and 72 hours after uniform cell plating (5000 cells), lysed, and “reagent” was added, and DNA quantity measured by spectrophotometry. **C.** Measurement of Ly2 cell proliferation between MK2 parental and KO cell lines *in vitro* via Cyquant proliferation assay. D. Kill curve of PF3644022 on Ly2 cells in culture using CyQuant to determine the number of remaining cells after 48 hours.

Supplemental Figure 3

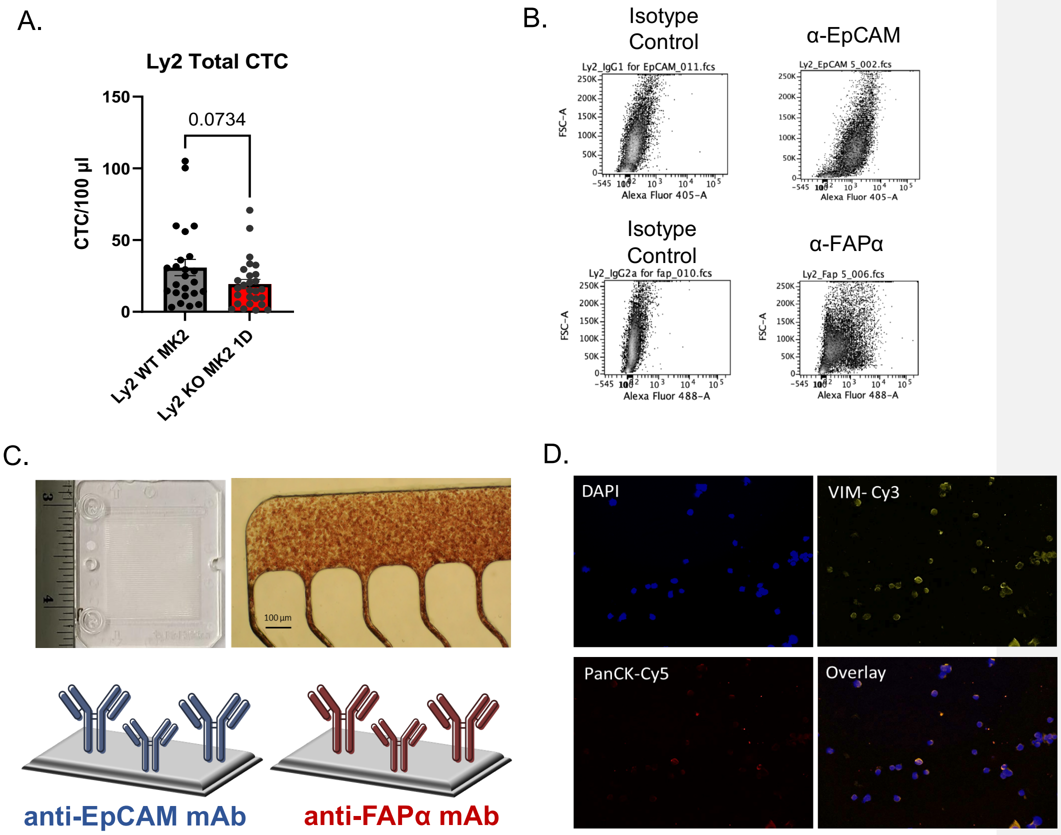

Supplemental Figure 3. Ly2 Expresses both EpCAM and FAPα. A. Pooled data for 2 experiments examining number of CTCs between Ly2 MK2 WT and Ly2 MK2 KO tumors. B. Ly2 tumors underwent flow cytometry to determine extent of FAPα expressing and EpCAM expressing cells. Appropriate isotype controls for both α-EpCAM and α-FAPα were used. Percent cell expression was analyzed using FloJo. **C.** Mice were terminally exsanguinated via cardiac puncture and blood stored with EDTA. Blood was passed first through an anti-FAPα microfluidics device followed by passage through an anti-EpCAM microfluidics device where Ly2 tumor cells expressing the appropriate surface marker are pulled out. **D.** Once captured cells were thoroughly washed, cells were eluted off the devices and enumerated. Cells underwent immunophenotyping by immunofluorescence where cells were stained using Vimentin-Cy3 (mesenchymal marker) and PanCK-Cy5 (epithelial cell type marker) and counterstained with DAPI (nuclear marker).

Supplemental Figure 4.

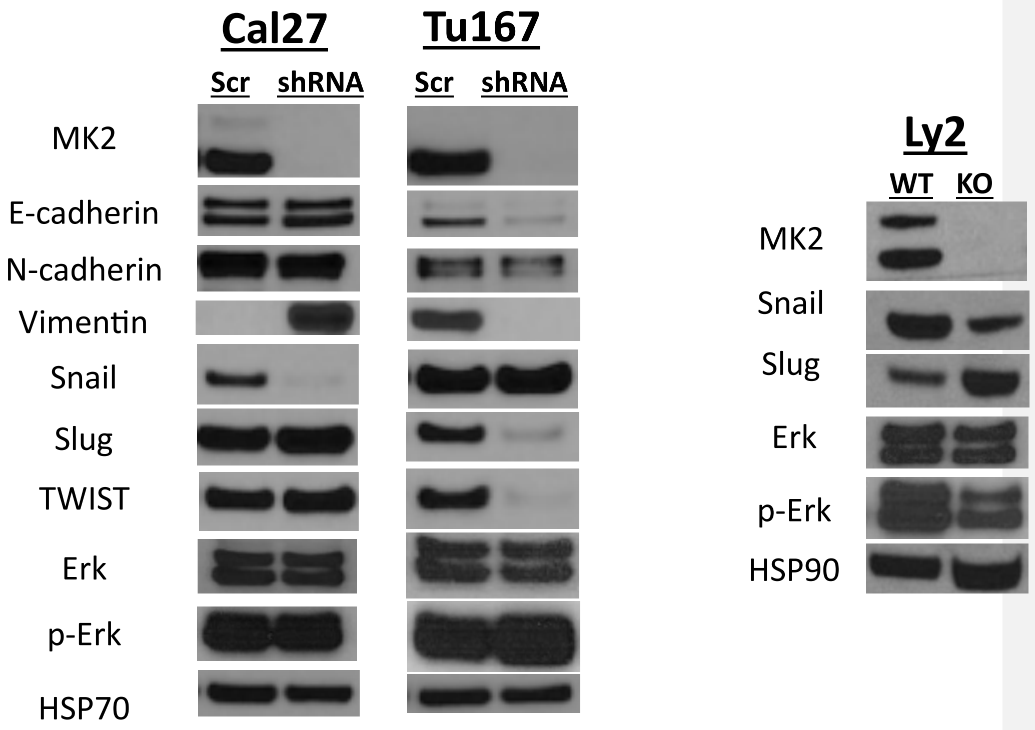

Supplemental Figure 4. Epithelial to mesenchymal protein expression A. Western blot data for 3 different cell lines showing the expression of EMT-related proteins and Erk signaling in MK2 knockdown/knockout cells. These blots are additional proteins that were visualized from the same samples as shown in figure 1 and Figure 2. MK2 bands are shown as reference.

Deleted: E
